# Deconstructing Complex Interventions: Piloting a Framework of Delivery Features and Intervention Strategies for the Eating Disorders in Weight-Related Therapy (EDIT) Collaboration

**DOI:** 10.3390/nu15061414

**Published:** 2023-03-15

**Authors:** Natalie B. Lister, Hiba Jebeile, Rabia Khalid, Samantha Pryde, Brittany J. Johnson

**Affiliations:** 1 Children’s Hospital Westmead Clinical School, The University of Sydney, Sydney 2145, Australia; 2 Institute of Endocrinology and Diabetes, The Children’s Hospital at Westmead, Sydney 2145, Australia; 3 Caring Futures Institute, College of Nursing and Health Sciences, Flinders University, Bedford Park, Adelaide 5042, Australia

**Keywords:** weight loss, diet interventions, intervention coding, obesity, eating disorders, behaviour change

## Abstract

(1) Background: weight-management interventions vary in their delivery features and intervention strategies. We aimed to establish a protocol to identify these intervention components. (2) Methods: a framework was developed through literature searches and stakeholder consultation. Six studies were independently coded by two reviewers. Consensus included recording conflict resolutions and framework changes. (3) Results: more conflicts occurred for intervention strategies compared to delivery features; both required the updating of definitions. The average coding times were 78 min (SD: 48) for delivery features and 54 min (SD: 29) for intervention strategies. (4) Conclusions: this study developed a detailed framework and highlights the complexities in objectively mapping weight-management trials.

## 1. Introduction

Behavioural interventions including diet, physical activity, and psychological components are first-line treatments for obesity [1,2,3,4]. However, there are ongoing concerns that behavioural weight management, or some included strategies or methods of delivery, may induce or exacerbate eating disorders [5,6,7]. The eating disorders in weight-related therapy (EDIT) Collaboration aims to identify individual changes in eating disorder risk during weight management; further details are available elsewhere [8,9]. We hypothesise that specific components of interventions may influence eating disorder risk. For example, dieting predicts eating-disorder development in community samples [10], and dieting at a “severe” level is considered risky in terms of the likelihood of triggering binge-eating episodes [11]. Hence, there is a need to examine interventional evidence to determine whether specific intervention components may increase or decrease eating-disorder risk.

Understanding how intervention components may increase or decrease the risk of eating disorders would enable future interventions to be optimised. To date, no systematic examination of weight-management intervention components relevant to eating disorder risk has been conducted. Evidence shows that multicomponent (dieting, physical activity and behavioural) interventions are, for most people, effective for weight loss in the short term [12,13]. Further, systematic reviews have demonstrated that some components of these complex interventions may be more effective than others. For example, certain behaviour-change techniques, encouragement for positive health-related behaviours and contact with a dietitian are associated with intervention success [14,15,16]. Such research provides a useful insight into the tailoring of weight-management interventions to improve effectiveness. However, no such evidence synthesis has examined the *risk* of weight-management interventions. Detailed mapping of weight-management intervention components is an important step in understanding how weight-management trials may increase or decrease eating disorder risk.

This study aimed to establish a detailed, objective coding framework of intervention components (i.e., delivery features and intervention strategies) used in weight-management interventions for the EDIT Collaboration.

## 2. Materials and Methods

### 2.1. Development of a Coding Framework

The coding framework was developed through an iterative consultation process. An initial list of intervention components was drafted (HJ) and refined (NBL and BJJ). The initial codes were then expanded and refined through consultation with experts in the field via the EDIT Collaboration Scientific Advisory Panel and Stakeholder Advisory Panel including clinicians, researchers and people with lived experience of obesity and/or eating disorders [8]. The revised coding framework was further refined via a stakeholder consultation survey, with input from researchers, clinicians and those with lived experience of obesity and/or eating disorders internationally [17]. Stakeholders were asked to rate intervention strategies for likelihood to increase or decrease eating disorder risk within the context of weight management, and to identify any additional strategies which may be relevant to eating disorder risk [17]. A detailed guidebook was developed which included a descriptor for each unique code.

Delivery features are defined as “*a broad number of intervention characteristics that relate to how an intervention is delivered*” [18]. Delivery features were developed based on the Template for Intervention Description and Replication (TIDieR) checklist [19], including the overarching goal, target population, materials provided, procedures used, who delivered the intervention, delivery mode, intervention setting and dose, as well as any tailoring, modifications and fidelity measures. We also summarised the number and range of different outcome assessment procedures, as these may unintentionally deliver important messages about the aim and intended outcome of the intervention in addition to the planned intervention content. Categories under each delivery feature item/cluster were developed for this project drawing on relevant examples from child obesity prevention [18] and the Human Behaviour Change Project ontologies [20,21].

Intervention strategies is the broad term used to describe the behaviour change content of interventions grouped under key categories (i.e., highest level grouping) relevant to weight-management interventions. Clusters (i.e., mid-level grouping) of intervention strategies were captured under the following categories: intervention intent, framing and outcomes, dietary strategies, eating behaviours/disorder eating, movement and sleep related strategies, and psychological health-related strategies. There were 86 unique intervention strategies (i.e., lowest-level grouping) across these five clusters.

### 2.2. Eligibility Criteria for the Pilot

Trials eligible for inclusion in the EDIT Collaboration were randomised controlled trials of behavioural weight-management interventions recruiting adolescents (aged 10 to <19 years at baseline) or adults (aged ≥18 years at baseline) with overweight or obesity defined as body mass index (BMI) z-score > 1 in adolescents and BMI ≥ 25 kg/m^2^ in adults [22]. Trials must measure eating disorder risk at baseline and post-intervention or follow-up using a validated assessment tool.

Purposeful sampling methods were used to select studies for piloting. Eligible studies (n = 73 as at May 2022) were grouped by decade of publication, with each group weighted by the total number of studies to determine how many studies should be selected from each decade. Both random and purposeful approaches guided the final selection of studies for piloting, ensuring diversity of target population (adolescents, adults), availability of a published protocol and study country. For pragmatic reasons, any studies identified that declined to join the EDIT Collaboration were replaced with a study of a similar profile.

### 2.3. Pilot Process

Published intervention descriptions, from trial registries, protocol and main results publications, were used to code intervention components using a standardised procedure following a brief training session. The training session involved familiarisation with the coding framework and practising coding to assist with consistency. Each unique intervention arm was coded by two independent coders (RK and SP, with a background in dietetics, and psychology, respectively), conflicts were identified and resolved through discussion (all authors). Duration of coding time was recorded.

Following initial coding and consensus of all studies, all authors critically discussed and reviewed the coding framework to ensure adequate coverage and clarity of delivery features and intervention strategies. Existing codes and descriptors were refined and additional codes included from discussion. Studies were then recoded using the updated codes.

### 2.4. Synthesis of Results

Descriptive statistics were used to summarise coding conflicts, number of modifications to the codebook, and to calculate the average and standard deviation (SD) of the time required to code components of each study. Results were synthesised separately for delivery features and intervention strategies, and examined by cluster.

## 3. Results

The characteristics of the selected studies are available in Supplementary Table S1. Six studies consisting of a total of 14 active intervention arms were selected. The included studies originated from the United States of America (n = 3), Australia (n = 1), Brazil (n = 1), and the United Kingdom (n = 1). The average time to code each study was 78 min (SD: 48) for delivery features and 54 min (SD: 29) for intervention strategies.

There was a greater number of coding conflicts in the intervention strategies (n = 237, 19.7%) compared to the delivery features (n = 156, 13.9%) (Table 1). The number of conflicts for each intervention arm ranged between 7 and 16 (8.9–20.5%) for delivery features and 2 and 34 (2.3–39.5%) for intervention strategies. When coding the delivery features, unclear definitions were the most common reason for conflicts and updating these definitions was required to achieve consensus. For instance, “duration of contact” was redefined to duration in minutes rather than brief, moderate or extended contact, which was subjective and difficult to code. In contrast, the consensus discussions for the intervention strategies revealed varying interpretations of definitions due to differing coder backgrounds. For example, the cluster “delivery of dietary intervention” had the greatest proportion of conflicts due to one coder having a comprehensive knowledge of clinical dietetic interventions. A total of 43 code definitions were modified, 15 new variables were added, and 7 were removed following the consensus meetings (Supplementary Table S2).

Consensus procedures for the new code resulted in 28 conflicts (13.3%) which were resolved through discussion and no further updates to variable definitions were required. The final revised coding framework included 86 delivery features (Table 2) and 88 intervention strategies (Table 3).

## 4. Discussion and Conclusions

The EDIT Collaboration aims to identify intervention components that increase or decrease eating disorder risk as part of weight-management interventions [9]. We developed a framework for identifying intervention components, by deconstructing complex interventions into well-defined delivery features and intervention strategies. Using an established coding framework reduces the subjectivity of intervention deconstruction. In addition, this pilot study demonstrates the resourcing required to conduct a comprehensive deconstruction of complex interventions.

Our study highlights the need for utilising established and tested definitions to appropriately deconstruct complex behavioural interventions. The taxonomic deconstruction of interventions is useful for examining which components of an intervention may be driving outcomes. However, previous studies of deconstruction behavioural weight-management interventions have predominately focussed on weight outcomes [14,15,16,18] or other measures of effectiveness [23,24,25]. Future research should consider whether interventions, or components of interventions produce unintended effects, such as increasing eating-disorder risk [5].

The strengths of this study include the use of a systematic coding process to validate the codes and definitions within the framework developed through extensive stakeholder consultation involving expertise from both the obesity and eating disorder fields. The framework was tested on a range of studies, varying in population (adolescents, adults), countries, and publication date. Coders were from differing disciplines (dietetics and psychology). The limitations include reliance on published or publicly available intervention descriptions, which may provide insufficient detail in the reporting of intervention components [26]. Further, we did not quantify the frequency or intensity of the intervention strategies.

Our coding framework will be implemented for all trials included in the EDIT Collaboration to examine eating-disorder risk during weight management [27]. Moreover, this framework can provide insight into a broad range of weight-management interventions and can be transferred or adapted to examine other safety or effectiveness outcomes (e.g., weight regain, health-related QOL, depression, etc). Coding frameworks, such as the one developed in this study, can assist in the transparent and systematic coding of existing interventions to enhance our understanding of the components of complex interventions.

## Figures and Tables

**Table 1:** Summary of conflicts across clusters of intervention components.

Category	Variables in Category (n)	Conflicts (n)	Proportion of Total Codes as Conflicts (%)	Common Reason(s) for Conflict
Delivery features ^a^				
Why—theory: rational, theory or goal	3	14	33.3	Definitions require clarification
Psychological theory or framework	10	9	6.4	Differing coder backgrounds
Target population/recipient of the intervention: age group	2	0	0.0	Definitions require clarification
Target population/recipient of the intervention: weight category	3	13	31.0	Definitions require clarification
Target population/recipient of the intervention: support	2	0	0.0	n/a
What—materials: physical or informational materials, including those provided to participants	13	18	9.9	Definitions require clarification
What—procedures: procedures, activities, processes used in the intervention	5	14	20.0	Definitions require clarification
What—outcome measures	7	13	13.3	Definitions require clarification
Who provided—intervention delivered by: personnel, training, qualifications	9	6	4.8	Definitions require clarification
Training for the interventionist	2	4	14.3	Definitions require clarification
How—delivery mode:	4	0	0.0	n/a
Individual/group	3	10	23.8	Definitions require clarification
Where—intervention setting: Location	9	20	15.9	Definitions require clarification
When and how much—intervention dose	3	23	54.8	Unclear definitions
Tailoring	1	4	28.6	Definitions require clarification
Modifications	2	2	7.1	Definitions require clarification
Fidelity	2	6	21.4	Unclear definitions; difficulty in coding with available resources
Total	80	156	13.9	
Intervention strategies				
Framing of the intervention (communication strategies)	5	24	34.3	Differing backgrounds
Outcome-related strategies	7	28	28.6	Required additional categories
Nutrition education	7	18	18.4	Differing coder backgrounds
Dietary monitoring	3	11	26.2	Differing coder backgrounds
Dietary prescription	7	27	27.6	Differing coder backgrounds
Delivery of dietary intervention	6	40	47.6	Differing coder backgrounds
Dietary behavour change strategies	6	13	15.5	Differing coder backgrounds
Addresses disordered eating	3	8	19.0	Definitions require clarification
Promotes healthful/helpful eating behaviours	6	5	6.0	Definitions require clarification
Physical activity education	5	5	7.1	Definitions require clarification
Physical activity prescription	4	12	21.4	Differing coder backgrounds
Physical activity monitoring	2	4	14.3	Differing coder backgrounds
Behaviour change strategies related to physical activity	4	6	10.7	Definitions require clarification
Addressing sedentary time	4	6	10.7	Definitions require clarification
Addressing sleep health	2	2	7.1	Definitions require clarification
Addresses mental health conditions, e.g., depression, anxiety, PTSD	4	12	21.4	Differing coder backgrounds
Addresses body image	3	2	4.8	Definitions require clarification
Addresses weight stigma	2	0	0.0	n/a
Psychosocial health-related monitoring	3	2	4.8	Definitions require clarification
Behaviour change strategies related to psychosocial issues	3	12	28.6	Differing coder backgrounds
Total	86	237	19.7	

^a^ Adapted from [19].

**Table 2:** Codebook of delivery features ^a^.

Category	Cluster	Components or Variables
Why—theory	Rationale, theory or goal	Weight maintenance—weight stabilisation but not weight loss
		Weight loss—intervention aims for decrease in body mass
		Weight loss and maintenance
	Psychological theory or framework underpinning the intervention	Cognitive behaviour therapy (CBT)
		Enhanced cognitive behaviour therapy (CBT-E)
		Acceptance and commitment therapy (ACT)
		Dialectical behaviour therapy (DBT)
		Family-based therapy (FBT) does not code family-based treatment of childhood obesity.
		Interpersonal therapy (IPT) for binge eating
		Trauma-informed care
		Compassion-focused therapy
		Motivational interviewing
		General theory name (not based on psychological theory)
Target population/recipient of the intervention	Age group	Adolescent
		Adult
	Weight category	Overweight
		Obesity
		Severe obesity
	Support	Individual
		Individual with support person/s, e.g., partner, parents (adolescents and parents separately or together as family-based approach)
		Family or household-based treatment approach
What	Materials: physical or informational materials, including those provided to participants	Information sheets/booklets (hardcopy resources)
		Food
		Diet monitoring materials
		Supplements (i.e., protein powder, vitamins)
		Meal-replacement products
		Sports equipment
		Fitness/activity tracker
		Body scales
		Food scales
		Mobile app
		Website access (online resources)
		Social media
		Other
	Procedures: procedures, activities, processes used in the intervention	Nutrition education
		Energy prescription or target
		Physical activity education
		Exercise classes
		Psychological component
	Outcome measures	Weight/adiposity
		Physical health outcomes
		Psychosocial/mental health outcomes
		Eating behaviour outcomes
		Individual weighing at visits
		Blind weighing at visits
		Group weighing
		Communication about the ability to decline weight or opt out of weighing during visits
Who provided or delivered the intervention	Personnel, training, qualifications	Dietitian/nutritionist
		Nurse
		Exercise physiologist/physiotherapist/personal trainer/other exercise professional
		Psychologist/counsellor
		Physician—paediatrician, GP, endocrinologist
		Pharmacist
		Researcher/non-health professional
		Self-delivered, e.g., self-help program (no researcher/health professional contact)
		Other
	Training for the interventionist	Intervention-specific training
How	Delivery mode	Face-to-face
		Computer/web-based/online
		Call (telephone, video)/SMS
		Printed material
	Individual/group	Individual
		Group
		Peer/social support, e.g., online forums, family participating in the intervention
Where	Intervention setting location	Hospital outpatient
		Hospital inpatient
		University
		Primary care (e.g., GP clinic, medical centre)
		Community
		School
		Household residence
		Virtual
		Commercial provider, e.g., WW, Jenny Craig, Slimming World
		Workplace
When and how much—support provided during the intervention	Intervention dose:	Total number of contacts
		Overall intervention duration (weeks)
		Intensity (frequency of contact: <weekly; 2–3 weeks; monthly; >monthly; staged approach (weekly to >monthly)
		Duration of contact/sessions (minutes)
	Post-intervention support	Referral to other services
		Additional information provided
	Tailoring	Was there an element of tailoring in the intervention
		What was the tailored/subgroup
Fidelity	Modifications	Was the intervention modified from plans
	Fidelity	Planned fidelity measures
		Actual fidelity measures

^a^ Adapted from [19].

**Table 3:** Codebook for intervention strategies.

Category	Cluster	Component or Variable
Intervention intent, framing and outcomes	Framing of the intervention (communication strategies)	Education provided on obesity as a disease
		Education that weight loss is required to improve health outcomes
		Education that health outcomes are not dependent on weight
		Education that health behaviours are linked to health outcomes
		Feedback on change in metabolic health outcomes (e.g., insulin sensitivity, cholesterol levels)
	Outcome related strategies	Encourages weight-focused goals
		Discourages weight-focused goals (instead focused on health-related goals)
		Feedback on weight change during the intervention
		Feedback on other measures of weight adiposity (e.g., body composition, waist circumference)
		Encourages self-monitoring of weight (e.g., self-weighing at home)
		Discourages home weighing or frequent weighing
		Promotes weight loss rewards or incentives
Dietary strategies	Nutrition education	Education on portion size (e.g., portion plate model, serving sizes, etc.)
		Education on label reading
		Education on metabolism
		Education on healthy-eating guide (e.g., promotes balanced meals and food groups)
		Education on energy/macronutrient (e.g., fat, sugar) content of foods
		Categorisation of foods as good versus bad (e.g., traffic light system; defines foods as good vs. bad (e.g., treat/sometimes foods))
		Provides cultural adaptations relating to diet
	Dietary self-monitoring	Dietary self-monitoring—food based (e.g., food diary, points system)
		Dietary self-monitoring—energy based (e.g., calorie counting)
		Dietary self-monitoring—weighing food
		Review/feedback on self-monitoring (e.g., feedback on food diary)
	Dietary prescription	Hypocaloric diet (reduced calorie diet)
		Traffic light diet (categorising foods as red, yellow, green)
		Intermittent energy restriction/intermittent fasting (chrononutrition)
		Macronutrient prescription (e.g., low carbohydrate, high protein)
		Ketogenic diet
		Very low energy diet (VLED/VLCD—restrictive calorie restriction, e.g., 800–1000 kcal/day)
	Delivery of dietary intervention	Prescriptive/specific meal plan (external control)
		Flexible meal plan (provides choice, ownership over dietary intake)
		Use of meal replacement products—partial or full
		Promotes “free” foods, ad-lib intake of certain foods
	Dietary behavour change strategies	Problem solving the barriers to dietary change
		Feedback on dietary behaviours (e.g., diet history at visits)
		Encourages dietary-focused goals with/without review
		Shopping support (planning, product choice, family/partner involvement in food purchases)
		Addresses home/food environment (e.g., identifying triggers, permissive vs. restrictive environment; stimulus control)
		Addresses food/meal preparation skills (e.g., cooking demonstrations, recipes)
Eating behaviours/disordered eating	Addresses disordered eating	Identifies disordered eating behaviours (e.g., binge eating, emotional eating, secret eating, guilt related to eating, loss of control over eating)
		Explores individual underlying causes/drivers of disordered eating (e.g., teasing/bullying, trauma, body image disturbance/preoccupation with weight and shape, emotional regulation)
		Addresses disordered eating behaviours and cognitions (e.g., identifying triggers, strategies to prevent emotional eating, over-focus on energy expenditure)
		Education on risk of eating disorders
	Promotes healthful/helpful eating behaviours	Promotes mealtime routines (e.g., regular meals, avoid meal skipping)
		Promotes meal time support (e.g., support while eating, family meals, social eating)
		Encourages mindful-eating principles or practice (e.g., avoiding distractions while eating)
		Encourages intuitive eating principles or practice (e.g., promotes anti-diet, hunger and fullness, food enjoyment, body respect)
		Addresses meal time environment
		Increasing awareness of hunger/fullness/satiety
Movement and sleep-related strategies	Physical activity education	Education to increase physical activity (e.g., staged introduction of activity, suggested activities)
		Promotes joyful movement and activity
		Encourages strict/formal activity plan (e.g., gym program)
		Education on non-exercise activity thermogenesis (NEAT) (energy expended during tasks of daily living)
		Provides cultural adaptations relating to physical activity
	Physical activity prescription	Provides a prescriptive exercise plan
		Provides flexible exercise plan (e.g., suggested activities, encouraging choice)
		Provides supervised group exercise classes/program
		Provides individual personal training
	Physical activity monitoring	Self-monitoring of activity (e.g., diary, pedometer)
		External feedback on self-monitoring (e.g., feedback on exercise diary, step count)
	Behaviour-change strategies related to physical activity	Encourage activity-focused goals (including time, duration, mode) with/without review
		Increasing skills to undertake physical activity (e.g., demonstration of activity such as pictures/videos/live demos, help with scheduling/planning for activity)
		Feedback on physical activity behaviours and/or change in fitness
		Problem solving the barriers to physical activity
	Addressing sedentary time	Education to reduce/limit sedentary time (e.g., screen time)
		Sedentary-time-focused goals (time, duration, mode) with/without review
		Encourages self-monitoring of sedentary time (e.g., screen time monitoring, setting app limits, reminders to stand)
		Problem solving the barriers to reducing sedentary time
	Addressing sleep health	Education on sleep health (e.g., duration, quality, routine)
		Sleep-health-focused goals with/without review
		Encourages self-monitoring of sleep (e.g., sleep diary)
		Problem solving the barriers to improving sleep health
Psychosocial health-related strategies	Addresses mental health conditions, e.g., depression, anxiety, PTSD	Identifies mental health condition
		Provides referral for psychological support
		Addresses mental health condition within the intervention
		Addresses self-esteem
	Addresses body image	Addresses body image concerns
		Education on the role of social media (e.g., media-literacy training)
		Promotes body compassion/acceptance/positivity
	Addresses weight stigma	Education and/or strategies to increase resilience to weight stigma, bullying, teasing
		Addresses weight-focused communication skills (e.g., how to communicate with peers/family about weight, how to address weight-related comments from peers/family)
		Education to support network persons on weight stigma/teasing
	Psychosocial health-related monitoring	Self-monitoring of thoughts, feelings, mood (e.g., mood diary)
		Review/feedback on self-monitoring (e.g., mood diary)
		Encourages self-assessment of overall wellbeing (e.g., reflective practice)
	Behaviour change strategies related to psychosocial issues	Encourages psychosocial health-related goals with/without review
		Increases skills to manage psychosocial health (e.g., stress management)
		Inclusion of peer-/social-support strategies

## Data Availability

Not applicable.

## Supplementary Materials

The following supporting information can be downloaded at: https://www.mdpi.com/article/10.3390/nu15061414/s1, Table S1: Characteristics of studies included in pilot coding; Table S2: Modifications to coding framework [28,29,30,31,32,33].
