# Supplementary material for: Deconstructing Complex Interventions: Piloting a Framework of Delivery Features and Intervention Strategies for the Eating Disorders in Weight-Related Therapy (EDIT) Collaboration
Source: Nutrients. 2023 Mar 15;15(6):1414. doi: 10.3390/nu15061414 (PMC10056322; doi:10.3390/nu15061414)
Supplement: Supplementary file 1 [file nutrients-15-01414-s001.zip › nutrients-2240896-supplementary.pdf]

Supplementary Table 1: Characteristics of studies included in pilot coding

Study	Year	Country	Population	Intervention arms
Lister et al [1]	2020	Australia	Adolescents	1. Intermittent energy restriction
				2. Continuous energy restriction
Salvo et al [2]	2018	Brazil	Adults	1. Treatment as usual (TAU)
				2. Mindfulness eating awareness (MBEAT) + TAU
				3. Mindfulness health promotion (MBHP) + TAU
Beaulieu et al [3]	2019	United Kingdom	Adults	1. Intermittent energy restriction
				2. Continuous energy restriction
Di Marco et al [4]	2009	United States of America	Adults	1. Guided self-help behavioural weight loss
				2. Guided self-help + motivational interviewing
Raynor et al [5]	2005	United States of America	Adults	1. Reduced snack variety
				2. No variety limit for snacks
Epstein et al [6]	2001	United States of America	Adolescents	1. Problem-solving skills for child
				2. Problem-solving skills for child and parent
				3. No additional problem-solving skills

Supplementary table 2: Modifications to coding framework

New delivery features added	
**Cluster**	**Variable**
Why – theory: Rational, theory or goal	Weight loss and maintenance
Target population/ recipient of the intervention: support	Family or household based treatment approach
What – outcome measures:	Eating behaviour outcomes
Where – intervention setting: Location	Virtual
When and how much – intervention dose:	Intensity
Post-intervention support	Referral to other sources
Post-intervention support	Additional information provided
**Removed delivery features**	
**Cluster**	**Variable**
Why – theory: Rational, theory or goal	Weight-neutral – intervention focus on health improvement (e.g. HAES approach)
**New intervention strategies**	
**Cluster**	**Variable**
Addresses disordered eating	Education on risk of eating disorders
Addressing sleep health	Education on sleep health (e.g. duration, quality, routine)
Addressing sleep health	Sleep health focused goals with/without review
Addressing sleep health	Encourages self-monitoring of sleep (e.g. sleep diary)
Addressing sleep health	Problem solving barriers to improving sleep health
Addresses weight stigma	Education to support network persons on weight stigma/teasing
Outcome related strategies	Feedback on change in metabolic health outcomes (e.g., insulin sensitivity, cholesterol levels)
Dietary self-monitoring	Dietary self-monitoring - weighing food
**Removed intervention strategies**	
**Cluster**	**Variable**
Dietary prescription	Moderate energy restriction (moderate calorie restriction)
Delivery of dietary intervention	Individual approach to dietary change
Delivery of dietary intervention	Family-oriented approach to dietary change
Addressing sleep health	Promotes regular sleep routine
Addressing sleep health	Promotes positive sleep environment (e.g. limit screen time before bed)
Framing of the intervention (communication strategies)	Language used during the intervention (e.g., weight loss, weight management, weight control, healthy lifestyle)

References

1. Lister, N.B., et al., *Fast track to health-Intermittent energy restriction in adolescents with obesity. A randomised controlled trial study protocol.* Obesity research & clinical practice, 2019.
2. Salvo, V., et al., *Mindfulness as a complementary intervention in the treatment of overweight and obesity in primary health care: study protocol for a randomised controlled trial.* Trials, 2018. **19**(1): p. 277.
3. Beaulieu, K., et al., *Matched Weight Loss Through Intermittent or Continuous Energy Restriction Does Not Lead To Compensatory Increases in Appetite and Eating Behavior in a Randomized Controlled Trial in Women with Overweight and Obesity.* The Journal of Nutrition, 2019. **150**(3): p. 623-633.
4. DiMarco, I.D., et al., *The use of motivational interviewing techniques to enhance the efficacy of guided self-help behavioral weight loss treatment.* Eating Behaviors, 2009. **10**(2): p. 134-136.
5. Raynor, H.A., H.M. Niemeier, and R.R. Wing, *Effect of limiting snack food variety on long-term sensory-specific satiety and monotony during obesity treatment.* Eating Behaviors, 2006. **7**(1): p. 1-14.
6. Epstein, L.H., et al., *Changes in eating disorder symptoms with pediatric obesity treatment.* The Journal of pediatrics, 2001. **139**(1): p. 58-65.
